# Gaze-Speech Coordination During Narration in Autism Spectrum Disorder and First-Degree Relatives

**DOI:** 10.3390/brainsci16010107

**Published:** 2026-01-19

**Authors:** Jiayin Xing, Joseph C. Y. Lau, Kritika Nayar, Emily Landau, Mitra Kumareswaran, Marcia Grabowecky, Molly Losh

**Affiliations:** 1 Department of Child and Adolescent Psychiatry, Child Study Center, Hassenfeld Children’s Hospital at NYU Langone, New York, NY 10016, USA or jiayinxing2018@u.northwestern.edu (J.X.); kritika.nayar@nyulangone.org (K.N.); 2 Roxelyn and Richard Pepper Department of Communication Sciences and Disorders, Northwestern University, Evanston, IL 60201, USA; josephcylau@northwestern.edu (J.C.Y.L.); emily.landau@northwestern.edu (E.L.); mitra.kumareswaran@northwestern.edu (M.K.); 3 Department of Psychology, Northwestern University, Evanston, IL 60201, USA; grabowecky@northwestern.edu

**Keywords:** ASD, gaze-speech coordination, narrative, family study, pragmatic language

## Abstract

**Background/Objectives:** Narrative differences in autism spectrum disorder (ASD) and subtle and parallel differences among their first-degree relatives suggest potential genetic liability to this critical social-communication skill. Effective social-communication relies on coordinating signals across modalities, which is often disrupted in ASD. Therefore, the current study examined the coordination of fundamental skills—gaze and speech—as a potential mechanism underlying narrative and broader pragmatic differences in ASD and their first-degree relatives. **Methods:** Participants included 35 autistic individuals, 41 non-autistic individuals, 90 parents of autistic individuals, and 34 parents of non-autistic individuals. Participants narrated a wordless picture book presented on an eye-tracker, with gaze and speech simultaneously recorded and subsequently coded. Time series analyses quantified their temporal coordination (i.e., the temporal lead of gaze to speech) and content coordination (i.e., the amount of gaze-speech content correspondence). These metrics were then compared between autistic and non-autistic groups and between parent groups and examined in relation to narrative quality and conversational pragmatic language skills. **Results:** Autistic individuals showed reduced temporal coordination but increased content coordination relative to non-autistic individuals with no significant differences found between parent groups. In both autistic individuals, and parent groups combined, increased content coordination and reduced temporal coordination were linked to reduced narrative quality and pragmatic language skills, respectively. **Conclusions:** Reduced temporal and increased content coordination may reflect a localized strategy of labeling items upon visualization. This pattern may indicate more limited visual, linguistic, and cognitive processing and underlie differences in higher-level social-communicative abilities in ASD. To our knowledge, this study is the first to identify multimodal skill coordination as a potential mechanism contributing to higher-level social-communicative differences in ASD and first-degree relatives, implicating mechanism-based interventions to support pragmatic language skills in ASD.

## 1. Introduction

Human social-communication is a multimodal system, where different component competencies collaborate to support successful communication [1]. Differences in the coordination of social-communicative signals in a socially informative manner have been observed in individuals with autism spectrum disorder (ASD) [2,3] and may relate to social-communication patterns that are characteristic of the diagnosis [4]. The current study examined social-communicative coordination in ASD, with a focus on differences in narrative, or storytelling, and investigated the coordination of two fundamental skills related to narration, i.e., gaze and speech [5], as the potential mechanism underlying narrative and broader pragmatic language differences. In this context, gaze was used as a measure of visual attention directed toward relevant story elements when viewing a storybook or a movie scene; speech refers to the coded verbal narratives obtained from the participants who described the story elements; and gaze-speech coordination refers to how visual information is integrated into speech and language to support storytelling [6,7,8,9,10,11,12]. Further, previous family studies demonstrated subtle but parallel subclinical social-communicative differences in first-degree relatives of autistic individuals (i.e., parents and siblings), suggesting potential genetic liability [13,14,15,16]. Therefore, the current study examined gaze-speech coordination during narration in both ASD and first-degree relatives of autistic individuals as a potential underlying mechanism of higher-level differences in social-communication skills. These skills include narrative abilities, i.e., organizing events in temporal and causal frameworks to communicate one’s reality during social interactions [17,18], as well as conversational skills, which involve managing dynamic social exchanges in real time.

Social-communicative coordination refers to the integration of different modalities to support communication. These social-communicative behaviors can complement each other to support communicative functions (e.g., mutual gaze during speech can communicate interest and intention) [19] and can coordinate temporally (e.g., gestures can augment speech by emphasizing specific information) [20]. This ability emerges in early child development (e.g., the coordination between reaching and gaze behaviors) [21,22] and is universally observed across cultures [1]. In individuals with ASD, cortical connectivity differences may lead to a perseverative cognitive style, reduced flexibility, and difficulty integrating information across modalities [23,24]. Indeed, studies have documented developmental differences in social-communicative coordination in ASD [2,3], the disruptions in which can further negatively impact social and language development [3,25,26]. These differences in social-communicative coordination within complex communicative contexts (e.g., narrative and conversation) may further serve as mechanistic contributors to high-level social-communication differences in ASD, such as pragmatic language abilities. However, this association between coordination differences and higher-order communication skills has not been examined in prior work on ASD.

The current study examines such relationships in autism and in parents in the context of narrative for the first time, which constitutes an important social-communication ability. Specifically, differences in the coordination of two component skills of narration—gaze and speech—were examined as potential contributors to narrative differences and broader social-communicative differences in ASD. Two primary types of gaze-speech coordination include temporal coordination (i.e., the extent to which gaze precedes speech on the same stimuli) and content coordination (i.e., the degree of correspondence between gaze focus and speech content) [12,27].

### 1.1. Gaze-Speech Temporal Coordination

Gaze-speech temporal coordination is the time lag between gaze and speech on the same visual stimulus. It may reflect underlying cognitive and speech and language planning processes and relate to higher-level social-communicative skills [5]. Temporal coordination has been studied in tasks such as rapid automatized naming (RAN) and narrative contexts [6,10,11,12].

The RAN task developed by Denckla and Rudel [28] assesses the speed of naming familiar visual stimuli, including letters, numbers, colors, and objects. Some studies recorded gaze during RAN to examine potential mechanisms of verbal output [5], offering a more nuanced examination of automaticity than traditional metrics of reaction time and error rate. As such, RAN may serve as a model to study gaze-speech temporal coordination. More specifically, temporal coordination during RAN reflects the fluency of underlying cognitive processes (e.g., visual processing and working memory) and linguistic processes (e.g., phonological retrieval and articulatory planning) that support complex speech and language skills, including reading [8,29,30,31,32,33].

Unlike the RAN task, a narrative context involves enriched visual information about story plots and characters (including both social and non-social information), higher-level linguistic processing (e.g., planning sentence structure within unfolding discourse), and integrative cognitive processes, such as interpreting relationships among story elements and the overarching story theme. In narrative tasks, participants narrated from a picture or storybook presented on an eye-tracker while gaze and speech were recorded simultaneously [8,9,12]. In typical development, two stages are observed: broad scanning to extract the gist, followed by focused gaze on individual objects to describe them in speech [9,12]. This sequence allows visual-conceptual processing and speech planning, supporting enriched and cohesive narratives [27,34,35,36]. The current study extends prior work by investigating temporal coordination during narration in autistic individuals and their first-degree relatives. This approach can shed light on related visual and cognitive processing patterns during narration and their potential impact on ASD-related differences in narrative quality and pragmatic language more broadly.

### 1.2. Gaze-Speech Content Coordination

In addition to temporal coordination, content coordination refers to the degree of content consistency or correspondence between what is fixated in gaze and what is described in speech during narration. This alignment suggests that gaze may be used strategically to enrich narrative content. In typical development, visual attention to contextual elements correlates with verbal descriptions of those elements, suggesting strategic use of gaze to enrich narrative content [27,34,35,36,37]. However, prior studies to date have not quantified content coordination in ASD, which may elucidate mechanisms underlying narrative differences.

### 1.3. Gaze-Speech Coordination in First-Degree Relatives

Previous studies have revealed a constellation of subclinical features that are parallel in quality to the defining characteristics of ASD in first-degree relatives of autistic individuals (i.e., parents and siblings), including differences in narrative functions and pragmatic language [13,14,15,16,38,39,40,41,42,43,44]. These features, called the broad autism phenotype (BAP), may reflect the genetic risk for autism [15,16]. Beyond behavioral and language features, parallel differences in underlying gaze-speech temporal coordination in RAN have been found in siblings and parents of autistic individuals [5,44], compared to non-autistic individuals and parent controls, respectively, suggesting similar mechanisms contributing to differences in higher-level social-communicative skills in both ASD and first-degree relatives.

### 1.4. The Current Study

This study examines gaze-speech temporal and content coordination during narratives in autistic individuals and their parents. The study additionally examines how these coordination processes may relate to differences in narrative quality and broader pragmatic language ability. We predict that autistic individuals would differ in both temporal and content gaze-speech coordination, with similar but subtler differences in their parents. We also expect that reduced gaze-speech coordination would be associated with reduced-quality narratives and conversational pragmatics in both groups.

## 2. Materials and Methods

### 2.1. Participants

Participants included 35 autistic individuals (ASD group), 41 individuals without autism (non-ASD group), 90 parents of autistic individuals (ASD parent group), and 34 parents of individuals without autism (parent control group). Parent groups included both parents when possible (ASD parent group: n = 20 dyads; parent control group: n = 2 dyads). All participants spoke English as their first language and were recruited from local registries, clinics, and advocacy groups. ASD diagnostic status was confirmed or ruled out using gold standard tools, including the Autism Diagnostic Observation Schedule-Second Edition (ADOS-2) [45] and/or the Autism Diagnostic Interview-Revised (ADI-R) [46]. ASD calibratedseverity scores were also extracted from ADOS-2, including Overall, Social Affect, and Restricted and Repetitive Behaviors (RRB) [45,47]. The Broad Autism Phenotype Questionnaire (BAPQ) [48,49] was used to assess the highest level of education completed by parents as well as autistic and non-autistic individuals.

For all participants, those who were under the age of 10 and who had a verbal IQ lower than 80, measured by the Wechsler Intelligence Scale for Children—Third Edition (WISC-IV), Wechsler Abbreviated Scale of Intelligence (WASI), or the Wechsler Adult Intelligence Scale (WAIS)—Third or Fourth Editions [50,51,52], were excluded to ensure sufficient language ability to produce a complex narrative. Participants with a family history of ASD-related disorders (e.g., fragile X syndrome and tuberous sclerosis) were also excluded. See Table 1 for detailed demographic information.

### 2.2. Procedures

Participants viewed a 24 page wordless picture book, *Frog, Where Are You?* [53] presented on a Tobii T60 series eye-tracker (Tobii Technology AB, Danderyd, Sweden) with a resolution of 1280 × 1024 pixels. All participants were seated approximately 50–60 cm from the screen during eye-tracking tasks. The frog story is about a series of adventures that a boy and his pet dog went through when looking for their missing frog and has been widely used as a well-controlled narrative elicitation task among children and adults across different languages [54], numerous neurodevelopmental populations [55,56], and in both ASD and first-degree relatives [57]. Participants completed the task in a quiet, private space in their home, a reserved space (e.g., hotel), or in the laboratory, depending on the scheduling needs of participants. Only the participant and experimenter were present. Parents did not accompany children to prevent practice effects, and sessions were scheduled at convenient times. While participants narrated each page, narrative speech was recorded by an external microphone (Blue Snowball or Logitech USB Desktop Microphone) (see Lee et al., 2020 [8] for details). Gaze and narrative data reported in prior work [5,8,57,58] were used to support new analyses applied in the current study, including detailed hand-coding of visual attentional focus and speech content and computational time series analyses (see details below).

### 2.3. Existing Data Processing

#### 2.3.1. Transcription

Narratives were transcribed by transcribers trained to ≥80% word reliability and blind to diagnostic status using conventions in either Systematic Analysis of Language Transcripts (SALT) [59] or EUDICO Linguistic Annotator (ELAN) software version 5.8 [60]. A total of 18.5% of transcripts (n = 37), randomly selected by diagnosis and sex, were double transcribed for reliability. Overall word-level reliability was 95.86%, with 95.98% for parent controls, 96.93% for ASD parents, 95.11% for non-ASD controls, and 94.67% for ASD participants.

#### 2.3.2. Alignment

Trained coders, blind to diagnosis, marked utterance onsets and offsets using TextGrids in Praat [61] (https://www.fon.hum.uva.nl/praat/, accessed on 1 September 2022; version 6.0.29). The Forced Alignment and Vowel Extraction [62] program was subsequently used to align speech with transcription at the word level automatically. The onset and offset times of speech during each word were further extracted using a Praat script (see Patel et al., 2020 [43] for details).

#### 2.3.3. Gaze Processing

Gaze data has been reported and processed in prior work [8,57,58]. Areas of interest (AOIs) were pre-selected in Tobii Studio software (Version 3 2.1), including social story characters and non-social setting elements. Fixations were defined based on the I-VT fixation filter available in Tobii Studio. Participants with a word-to-tracked eye movement time ratio greater than four words/second within each story episode (i.e., setting, searching, and resolution) were excluded, indicating poor gaze tracking during vocalization (see Lee et al., 2020 [8] for details).

### 2.4. Narrative Quality

Narrative quality was assessed using a hand-coding scheme developed by prior studies [57,58]. Primary measures of narrative quality included Affect/Cognition, which represents the percentage of descriptions of thoughts/emotions of the story characters; Story Components Present, which measures the inclusion of key story elements in the narrative; and Causal Inferences, which refers to the percentage of causal explanations of story events and actions of the story characters (see Nayar et al., 2024 [57] for details). These data were reported in prior work [57,58] and were used here for correlational analyses.

### 2.5. New Data Processing

#### 2.5.1. Narrative Coding

Speech content was hand-coded at the word level to determine the specific story component referenced in each spoken word. The coding scheme included key story elements related to the thematic content of each page of the story, such as the main story characters (e.g., boy, frog, and dog) and setting elements (e.g., moon and woods). Coders were trained to ≥80% reliability and blind to diagnostic status. Twenty percent of transcripts (n = 40) were randomly selected by diagnosis and sex and double-coded, with an inter-rater reliability of 98.2%.

#### 2.5.2. Gaze Coding

AOIs included in the existing gaze processing were further filtered to include only those relevant to the story theme, following the same coding scheme used for narrative speech. When AOIs overlapped, fixations were assigned to the AOI with greater social relevance to the story context (e.g., social stimuli over non-social stimuli and protagonists over secondary story characters). For each fixation, the onset and offset times and the AOIs fixated were extracted. The coded narrative speech and gaze time series were then aligned at 0.2 s intervals. No participants were excluded due to alignment issues.

#### 2.5.3. Pragmatic Language Ability

The Pragmatic Rating Scale-School Age (PRS-SA) [63] and the Pragmatic Rating Scale (PRS) [64] were used to assess conversational pragmatic language abilities in ASD and non-ASD groups, and parent groups, respectively. Both the PRS and PRS-SA capture components of conversational pragmatic language, such as conversational management and nonverbal communication [65]. The PRS-SA is rated based on conversational components of the ADOS-2, while the PRS ratings are based on conversations from a life history interview, where examiners converse with parents on topics such as their childhood, marriage, family relationships, and profession, etc. Coders were trained to ≥ 80% reliability and were blind to group status. Part of the coded PRS-SA and PRS files were reported in prior work [65,66]. In the current sample, 31% (n = 17) of the PRS-SA files and 84% (n = 116) of the PRS files were double-coded for reliability check, with an inter-rater reliability at 76.10% and 83.52%, respectively. Discrepancies were resolved between reliability coders, and consensus codes were used for analyses.

### 2.6. Data Analysis

#### 2.6.1. Gaze-Speech Coordination: Diagonal Cross Recurrence Profiles (DCRP) Analysis

Given the continuous nature of multimodal behaviors during narration, we applied diagonal cross recurrence profiles (DCRP) analysis using the CRQA package(version 2.0.7 [67] in *R* (version 4.4.1 (2024-06-14)) to quantify the content and temporal coordination between speech and gaze across time. DCRP is a recurrence-based non-linear approach to computational time series analysis. It measures the shared dynamics of two coupled time series, which have been increasingly employed in the cognitive and social sciences [68,69]. The primary measures used to quantify gaze-speech coordination are described below (see Supplement and Figure S1 for detailed computational methods) as follows:

1. Recurrence rate (RR): Recurrence rate quantifies the proportion of recurrence/correspondence between two time series (i.e., gaze focus and narrative content examining the same story component/visual stimuli). In the current study, a higher RR indicates greater consistency between gaze focus and speech content, representing greater gaze-speech content coordination.
2. Recurrence rate peak (RRpeak): Recurrence rate peak measures the highest proportion of correspondence between gaze and speech during narration. A higher RRpeak represents a greater maximum amount of coordination across different time lags between time series, which refers to the greatest level of content coordination or consistency between gaze and speech across narration in the current study.
3. Qlos: Qlos measures the extent to which one behavioral time series leads the other, i.e., gaze leads speech on the same story components in time. A higher Qlos represents a greater temporal lead of gaze over speech, or a higher average extent of gaze-speech temporal coordination across narration.

#### 2.6.2. Sham Data Samples

Sham data samples were created following methods developed in prior studies [70,71] to serve as a statistical baseline. Sham data samples were created by shuffling the time sequences of gaze and speech time series (iterations n = 10,000), and randomly pairing gaze and speech data from different individuals across diagnostic groups. The sham data samples were generated separately for ASD and non-ASD groups, and for the parent groups, with sample sizes matched to the participant groups. Further, the mean values of the three coordination measures, i.e., RR, RRpeak, and Qlos, in the sham data samples were extracted from those in real participants to quantify the temporal and content coordination levels relative to the randomized level.

#### 2.6.3. Group Comparisons

A series of mixed-effects linear regression models were applied using the lmer package(version 1.1-38) [72] in R (version 4.4.1 (2024-06-14)) for group comparisons on coordination metrics. Both measures of temporal coordination (i.e., Qlos) and the measure of content coordination (i.e., RR and RRpeak) from DCRP analyses were compared, with averages from the sham groups extracted. All models included diagnosis (ASD vs. non-ASD or ASD parents vs. parent controls) and covariates (i.e., age, gender, VIQ, and duration of speech) as fixed effects and participants as random effects. Age, gender, and verbal IQ were controlled as covariates given their potential influences on narrative. The duration of speech was covaried because of its potential influence on gaze-speech content coordination across narration (i.e., the longer the narration, the greater the possibility of correspondence between gaze focus and speech content). Given the expected subtle differences in non-clinical parent groups, both significant and marginally significant group differences were reported.

#### 2.6.4. Correlations

Partial Pearson correlations examined the associations between main measures of gaze-speech coordination (i.e., Qlos and RR) and ASD symptom severity, narrative quality, and conversational pragmatic language abilities, with age, sex, VIQ, and duration of speech as covariates. Within-family associations between mothers and children were also examined. Only statistically significant correlations (*ps* < 0.05) are reported. Benjamini-Hochberg-adjusted *p*-values [73] were calculated with an FDR of 0.10, following prior work [8,74] in heterogeneous ASD and BAP samples, to balance the control of false positives with sensitivity to subtle, potentially meaningful effects in subclinical populations.

The correlations with ASD symptom severity extracted from ADOS-2 via calibrated severity scores, including domains of Overall, Social Affect, and Restricted and Repetitive Behaviors (RRBs), were conducted in the ASD group only. Correlations between gaze-speech coordination and narrative quality measures (i.e., Affect/Cognition, Story Components Present, and Causal Inferences) and conversational pragmatic language (measured by PRS-SA for ASD and non-ASD groups, and by PRS for parent groups) were conducted. The correlations were examined in both ASD and ASD parent groups individually and in ASD and non-ASD groups combined and parent groups combined, respectively, to understand the potential contributions of differences in gaze-speech coordination to differences in higher-level social-communicative skills in ASD, first-degree relatives impacted by ASD-related genetic risk, and the broader general population.

Further, within-family correlations in mother-child dyads were examined on coordination measures. Specifically, mother-child correlations were examined in families of autistic individuals only (n = 18), because of the low sample size of families of non-autistic individuals (n = 8). Correlations between father-child pairs were not conducted because of low sample size (n = 11 for ASD families and n = 4 for families of non-autistic individuals).

### 2.7. GenAI Use

During the preparation of this manuscript/study, the authors used ChatGPT (OpenAI, GPT-5, 2025) for the purposes of language refinement and improving clarity of expression. The authors have reviewed and edited the output and take full responsibility for the content of this publication.

## 3. Results

### 3.1. Group Comparisons

**Figure 1:**
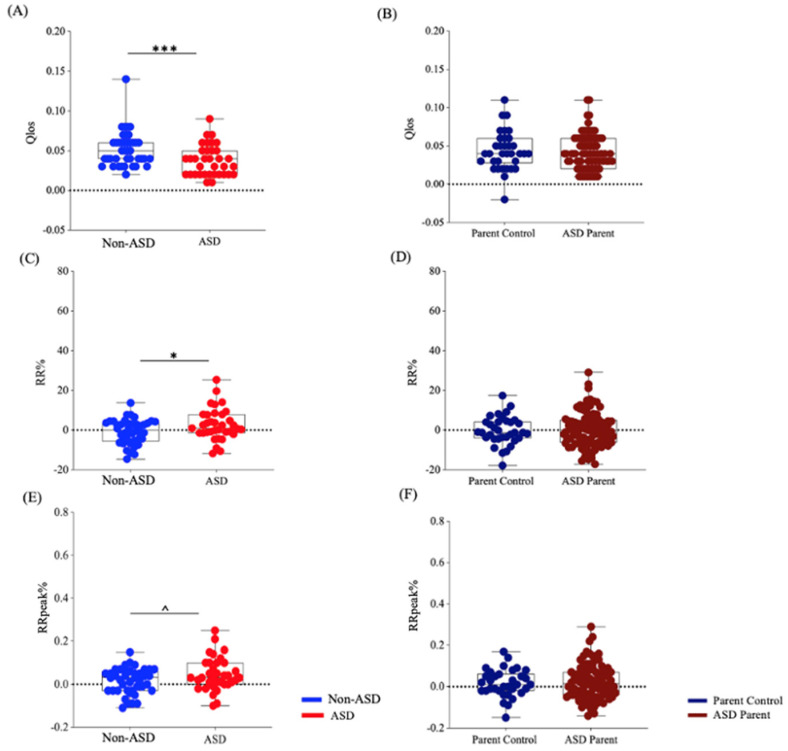
Gaze-speech Coordination Across Diagnostic Groups. Gaze-speech coordination measures: (**A**,**B**) Qlos: temporal coordination, the extent to which gaze was ahead of speech in time; (**C**,**D**) RR: content coordination, the total amount of content consistency between gaze and speech; (**E**,**F**) RRpeak: maximum content coordination across different time lags. The ASD group had reduced gaze-speech temporal coordination (**A**) and increased content coordination (**C**,**E**) compared to the non-ASD group, with no significant differences found in parent groups (**B**,**D**,**F**). Dashed lines represent the sham groups as the randomized control level. Box plots showed the five-number summary of a set of data, including the minimum, the 25th percentile, the median, the 75th percentile, and the maximum. ^ *p* < 0.10, * *p* < 0.05, *** *p* < 0.001.

For Qlos, the ASD group had reduced temporal coordination compared to the non-ASD group (*estimate* = −0.02, *t* = −3.81, *p* < 0.001, *Cohen’s d* = −2.98), whereas no significant difference was found between parent groups (*estimate* = 0.001, *t* = 0.21, *p* = 0.84, *Cohen’s d* = 0.13). For gaze-speech content coordination, the autistic group demonstrated increased content coordination measured by both RR (*estimate* = 4.19, *t* = 2.18, *p* = 0.03, *Cohen’s d* = 1.78) and RRpeak (*estimate* = 0.04, *t* = 1.96, *p* = 0.05, *Cohen’s d* = 1.6), compared to non-autistic individuals. No significant differences were found between parent groups for RR (*estimate* = 0.62, *t* = 0.35, *p* = 0.73, *Cohen’s d* = 0.22) or RRpeak (*estimate* = 0.008, *t* = 0.45, *p* = 0.66, *Cohen’s d* = 0.28) (see Figure 1). Effect sizes (*Cohen’s d*) are relatively large because *d* scales mean differences by the pooled standard deviation [75]. DCRP-derived gaze-speech coordination measures are continuous and temporally dense, which can result in reduced within-group variability; consequently, moderate absolute differences may yield large, standardized effects and should be interpreted in this measurement context.

### 3.2. Correlations with ASD Symptom Severity

Gaze-speech coordination was not significantly correlated with ASD symptom severity (see detailed statistics in Table S1 in Supplementary Materials).

### 3.3. Correlations with Narrative Quality

Greater gaze-speech content coordination, as measured by RR, was significantly correlated with increased inclusion of descriptions of emotional and cognitive states and behaviors in narrative storytelling (Affect/Cognition) in the ASD group (*r* = 0.40, *p* = 0.03, adjusted *p* = 0.34). In parent groups combined, a greater RR was associated with the inclusion of fewer key story elements and interactions among story characters (Story Components Present) (*r* = −0.19, *p* = 0.04, adjusted *p* = 0.34). No other significant correlations emerged (see detailed statistics in Table S2 in Supplementary Materials).

### 3.4. Correlations with Pragmatic Language Ability

**Figure 2:**
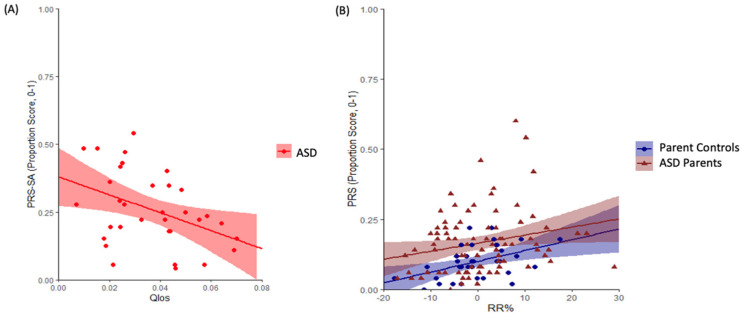
Correlations Between Gaze-Speech Coordination and Pragmatic Language Ability. (**A**) Reduced temporal coordination correlated with greater conversational pragmatic violations in the ASD group; (**B**) increased content coordination was associated with increased conversational pragmatic violations in parent groups combined. Shaded regions represent 95% confidence intervals.

Reduced temporal coordination between gaze and speech, as measured by Qlos, was correlated with more conversational pragmatic language violations, reflected by higher PRS-SA or PRS scores, in the autistic and non-autistic groups combined (*r* = −0.31, *p* = 0.03, adjusted *p* = 0.08). This finding was mainly driven by the ASD group (*r* = −0.37, *p* = 0.05, adjusted *p* = 0.09) (see Figure 2). In contrast, increased content coordination between gaze and speech, as measured by RR, was correlated with poorer conversational pragmatic language ability in parent groups combined (*r* = 0.26, *p* = 0.005, adjusted *p* = 0.04) (see Figure 2), which was driven by the ASD parent group (*r* = 0.26, *p* = 0.02, adjusted *p* = 0.08). No other significant correlations were found between gaze-speech content coordination and pragmatic language (see detailed statistics in Table S3 in Supplementary Materials).

### 3.5. Mother-Child Correlations

**Figure 3:**
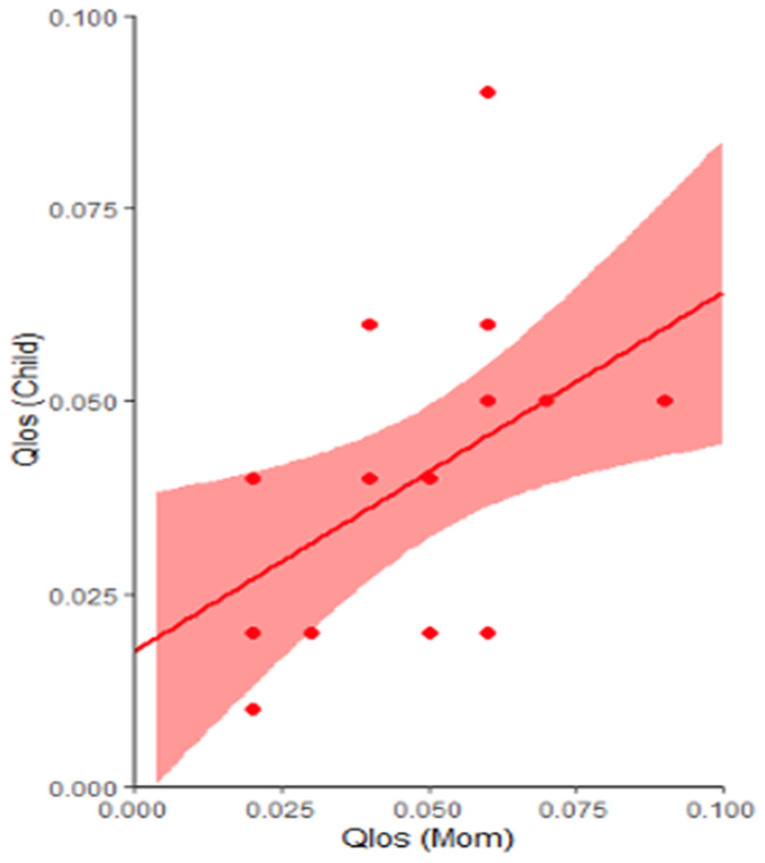
Mother-child Correlations for Gaze-Speech Temporal Coordination. Reduced temporal coordination in mothers was correlated with reduced temporal coordination in their children with ASD. Shaded regions represent 95% confidence intervals.

In the n = 18 mother-child dyads from ASD families, significant correlations were found for gaze-speech temporal coordination, such that reduced Qlos in mothers was correlated with reduced Qlos in their children with ASD (*r* = 0.57, *p* = 0.02, adjusted *p* = 0.03) (see Figure 3). No significant mother–child correlation was found for gaze-speech content coordination measured by RR.

## 4. Discussion

This study is the first to examine gaze-speech coordination during narration in autistic individuals and their parents. Two aspects of coordination were assessed as follows: (1) temporal coordination, representing the extent of gaze leading speech in time; and (2) content coordination, referring to the content consistency between gaze and speech. Reduced temporal and increased content gaze-speech coordination during narration were found in the ASD group compared to non-autistic controls. Although ASD parents showed a similar descriptive pattern—lower temporal coordination and higher content coordination compared to parent controls—these differences were more subtle and did not reach statistical significance, likely reflecting smaller effect sizes in subclinical populations. However, in both the ASD and ASD parent groups, differences in gaze-speech coordination were associated with differences in higher-level communicative abilities, including narrative and conversational pragmatic language abilities, providing potential insight into gaze-speech coordination as an underlying mechanism contributing to higher-level ASD-related social-communicative differences.

Reduced gaze-speech temporal coordination aligns with prior RAN studies showing shorter gaze-speech lag in autistic individuals [44]. These earlier results suggest reduced automaticity in the integration of visual and verbal information in ASD, a mechanism that may underlie narrative or higher-level social-communicative differences. The current study extends prior work from RAN tasks to a more complex and cognitively demanding linguistic context (i.e., narrative production) that mirrors language use in daily life and similarly observed reduced gaze-speech temporal coordination in ASD. Unlike RAN tasks, which involve simple visual and linguistic processing, the narrative task engages more enriched visual-conceptual processing of story plots and characters (both social and non-social information), high-level cognitive processes (e.g., extracting the gist of and relations within a scene, relating individual story elements to the overarching theme) before speech output, as well as higher-order executive-linguistic processing such as planning sentence structure within unfolding discourse [8,11,34]. Therefore, disrupted temporal coordination during narration in ASD may reduce the time available for visual, linguistic, and higher-level cognitive systems to operate in a coordinated manner and may serve as a potential mechanistic factor contributing to higher-order social-communicative differences in ASD.

The temporal coordination is precise, quantifiable, and neurocognitively grounded, moving beyond observable behaviors. The observed mother-child associations in temporal coordination suggest that gaze-speech coordination during narration may reflect heritable traits influenced by ASD-related molecular-genetic variability, consistent with prior literature on pragmatic language differences in the broad autism phenotype (BAP) [40,42,43,57]. Additionally, prior family studies have revealed potential patterns of maternal linearity, where phenotypic associations in social communication between mothers and autistic children appear more consistent and robust than those between fathers and autistic children, potentially reflecting a shared social-communicative “signature” [76]. By contrast, associations between fathers and autistic children appear to be more evident in the rigidity/repetitive behavior domain [5,76]. These mechanistic coordination differences may represent promising heritable features linking genetic likelihood to observable behaviors and could help reduce phenotypic heterogeneity in biological research by identifying more homogeneous family subgroups. In this context, the mother-child associations observed here may reflect shared genetic influences on the integrated visual, cognitive, and linguistic processes that support narrative communication, suggesting the potential value of temporal coordination as a window into the underlying mechanisms supporting social-communicative functioning in ASD and the BAP. Future studies of temporal coordination may benefit from a focus on genetically informed designs (e.g., twin or extended family studies) and longitudinal studies tracking the development of temporal coordination across early childhood to more explicitly examine temporal coordination as a potential phenotypic marker of heritable social-communication differences in ASD. Nevertheless, the current study may highlight the potential value of temporal coordination as a window into the underlying processes supporting social-communicative functioning in ASD and the BAP.

Although prior studies in non-autistic individuals suggest that gaze-speech content consistency reflects the integration of visual information to enrich speech and language production [27,35,37], the increased gaze-speech content coordination observed in ASD in the present study may reflect a different functional pattern. Specifically, heightened coordination may indicate a greater reliance on immediately available visual input during narration, biasing speakers toward directly labeling visually salient elements rather than integrating information across story components. This tendency may reduce inferences about relationships among events (e.g., cause-effect relations), characters, or the overarching narrative theme. This localized gaze-speech coordination pattern is consistent with Bayesian accounts of perception in ASD, which posit atypical weighting of prior expectations relative to sensory input, potentially resulting in greater reliance on moment-to-moment visual information and reduced integration of broader contextual cues [77].

An illustrative example highlights this distinction. When describing page 4 of the picture book, an individual with ASD stated, “the boy picked up his boot and looked inside and he turned over a stepstool and the dog stuck his head in the jar to try to find him there,” closely mirroring the sequence of visually fixated elements for this individual. In contrast, a non-autistic individual produced a higher-level summary—“he looked everywhere and the dog helped to look too”—that parsimoniously integrated the characters’ actions within the broader narrative theme of searching. A similar pattern emerged in descriptions of characters’ facial expressions. Individuals with greater gaze-speech content coordination tended to directly label emotional or cognitive states, potentially without fully integrating the contextual information or event relationships that give rise to those states. Accordingly, greater gaze-speech content coordination was associated with lower narrative quality, as reflected by fewer descriptions of interactions among key story elements and affective and cognitive states; however, these associations were significant only before multiple comparison correction and should therefore be interpreted cautiously. More broadly, a reliance on direct visual labeling may constrain interpretation of the wider social-communicative context, contributing to pragmatic language differences observed across both clinical and non-clinical populations, highlighting a potential shared cognitive or processing pattern between autistic individuals and their first-degree relatives.

Because the measures of content correspondence employed here (RR and RRpeak) capture gaze-speech content synchronization rather than a specific cognitive strategy, interpretations of increased coordination as reflecting overreliance on immediate visual input should require further investigation. Further, greater gaze-speech content coordination should not be interpreted as inherently maladaptive. Its functional significance is likely context-dependent. While heightened content coordination may be associated with reduced narrative integration in the present task, close alignment between visual attention and verbal output may support grounding, shared reference, and listener comprehension in other communicative contexts. Conversely, reduced gaze-speech coordination may also pose challenges, such as insufficient use of visual information to guide verbal output or weaker integration across perceptual and linguistic systems—patterns not captured by the current narrative paradigm. More broadly, effective communication may depend less on the absolute degree of synchrony than on the flexibility to dynamically shift between synchronous and asynchronous modes as tasks and social demands change. Future studies could therefore benefit from examining how both heightened and diminished gaze-speech coordination and the dynamics of changes in intrapersonal coordination may relate to social-communication skills across contexts.

Associations detected between gaze-speech coordination during narration and higher-level pragmatic language and social-communicative skills may also have important clinical implications for mechanism-based social communication interventions in ASD. Because gaze-speech temporal coordination may reflect the integration of visual, cognitive, and linguistic processes during narration, interventions that explicitly support multimodal integration, rather than focusing narrowly on labeling or describing visual information, may help strengthen the underlying mechanisms that contribute to cohesive storytelling and higher-level social-communicative skills. Evidence-based programs such as PEERS [78], which target pragmatic skills across specific conversational and social contexts (e.g., entering a conversation and being a good sport), may benefit from incorporating training in the coordination of gaze, speech, and cognitive processes to support effective narrative and conversational communication. Furthermore, speech-gaze coordination could serve as a mechanistic marker to track intervention progress, and future studies should further understand whether temporal coordination is malleable and can improve following social-pragmatics interventions.

These differences in gaze-speech temporal and content coordination observed in ASD may reflect variation in cortical structure and large-scale brain networks supporting visual, linguistic, and cognitive processes. Although neural mechanisms were not directly measured during narration, evidence from related paradigms (e.g., picture naming) suggests that coordination relies on interactions among distributed occipital, temporal, parietal, and frontal regions involved in visual processing, semantic integration, and speech planning [79,80,81,82,83]. Altered structure and function in these networks has been reported previously in ASD (e.g., angular gyrus, superior temporal sulcus, and Wernicke’s area [84,85,86,87,88]), which may contribute to reliance on compensatory pathways and differences in gaze-speech coordination. Future studies combining eye tracking and neuroimaging during narrative tasks are needed to directly examine how these distributed networks may support gaze-speech coordination and how such mechanisms may vary in autistic individuals and their first-degree relatives.

Several limitations of this study should be noted. First, although the frog story has been widely used to assess narrative ability across the lifespan, future work should examine its generalizability to real-world storytelling contexts. Moreover, while narrative is a primary form of social communication, conversations may provide a more naturalistic context mirroring language use during dynamic social interactions. Prior work examining gaze during conversational speech mainly focused on its social-pragmatic functions (e.g., turn-taking [89,90]), but little is known about gaze content and temporal coordination with speech in conversation. Therefore, future studies may leverage novel technology, such as head-mounted eye trackers, to examine gaze-speech coordination during conversations and its contributions to social communication. Second, the current study examined content and temporal coordination across the entire narration without a more detailed investigation into gaze patterns across time or the dynamics in gaze-speech coordination. Prior work has found that during narration, individuals may have a brief apprehension phase, or a global scan of pictures first, and then have a closer examination of each component closely [7]. Future studies might apply more fine-grained time course analyses to examine how gaze focus and its coordination with speech may dynamically change across time. Finally, due to the linguistic demands of the task, the current autistic sample did not include those with lower verbal or cognitive abilities. Future studies should include more diverse autistic samples, including individuals with higher support needs and larger groups of females, to better understand gaze-speech coordination across the spectrum.

## 5. Conclusions

In conclusion, the current study revealed differences in temporal and content coordination between gaze and speech during narration in autistic individuals. Associations between measures of coordination and narrative and conversational skill in both ASD and in parents implicate variability in gaze-speech as a potential mechanistic contributor to higher-level social-communication abilities. Future research should examine whether gaze-speech coordination is malleable and can improve following targeted social-pragmatics interventions, potentially serving as a mechanistic marker of intervention progress. Additionally, evidence-based programs, such as PEERS, could incorporate training in gaze-speech coordination to strengthen the underlying mechanisms of social communication.

## Figures and Tables

**Table 1:** Demographic Information.

	ASD	Non-ASD	ASD Parents	Parent Controls
	M (SD)	M (SD)	M (SD)	M (SD)
N (M/F) ^a^	35 (29/6)	41 (20/21)	90 (32/58)	34 (17/17)
Age (years) ^b^	18.79 (7.54)	18.70 (5.23)	45.59 (8.4)	41.39 (10.04)
FSIQ ^a,b^	106.51 (13.42)	117.59 (12.93)	109.76 (11.87)	117.03 (11.96)
VIQ ^a,b^	107.31 (14.88)	119.05 (12.39)	107.97 (12.09)	114.13 (13.06)
PIQ ^a,b^	104.40 (16.36)	113.21 (14.72)	109.34 (11.76)	115.90 (11.97)

^a^ Significant difference between ASD and non-ASD groups; ^b^ Significant difference between ASD parent and parent control groups. *Note*. FSIQ, VIQ, and PIQ refer to full-scale IQ, verbal IQ, and performance IQ derived from the Wechsler Abbreviated Scale of Intelligence, Wechsler Adult Intelligence Scale—Third or Fourth Editions, or the Wechsler Intelligence Scale for Children—Fourth Edition.

## Data Availability

The data that support the findings of this study are available from the corresponding author upon reasonable request due to privacy and ethical restrictions.

## Supplementary Materials

The following supporting information can be downloaded at: https://www.mdpi.com/article/10.3390/brainsci16010107/s1, Figure S1: Gaze-speech coordination was calculated based on the DCRP plots; Table S1: Correlations with ASD symptom severity; Table S2: Correlations with narrative quality; Table S3: Correlations with pragmatic language ability.

## Abbreviations

The following abbreviations are used in this manuscript:

ADI-R	Autism Diagnostic Interview—Revised
ADOS-2	Autism Diagnostic Observation Schedule, Second Edition
AOIs	Areas of Interest
ASD	Autism Spectrum Disorder
BAP	Broad Autism Phenotype
DCRP	Diagonal Cross Recurrence Profiles
ELAN	EUDICO Linguistic Annotator
FSIQ	Full-Scale Intelligence Quotient
IQ	Intelligence Quotient
LOS	Gray Line of Synchrony
PIQ	Performance Intelligence Quotient
PRS	Pragmatic Rating Scale
PRS-SA	Pragmatic Rating Scale—School Age
RAN	Rapid Automatized Naming
RR	Recurrence Rate
RRBs	Restricted and Repetitive Behaviors
RRpeak	Recurrence Rate Peak
SALT	Systematic Analysis of Language Transcripts
STS	Superior Temporal Sulcus
VIQ	Verbal Intelligence Quotient
WAIS	Wechsler Adult Intelligence Scale—Third or Fourth Editions
WASI	Wechsler Abbreviated Scale of Intelligence
WISC-IV	Wechsler Intelligence Scale for Children—Fourth Edition
