# Supplementary material for: Gaze-Speech Coordination During Narration in Autism Spectrum Disorder and First-Degree Relatives
Source: Brain Sci. 2026 Jan 19;16(1):107. doi: 10.3390/brainsci16010107 (PMC12839432; doi:10.3390/brainsci16010107)
Supplement: Supplementary file 1 [file brainsci-16-00107-s001.zip › brainsci-3888504-supplementary.pdf]

Methods: Gaze-speech coordination: diagonal cross recurrence profiles (DCRP) analysis

Given the continuous nature of multimodal behaviors during narration, we applied diagonal cross recurrence profiles (DCRP) analysis to quantify the content and temporal coordination between speech and gaze across time. DCRP is a recurrence-based non-linear approach of computational time series analyses. It measures the shared dynamics of two coupled time series, which have been increasingly employed in the cognitive and social sciences [51, 52]. 'Recurrence' refers to the 'synchrony' or 'coordination' between coupled time series [52], which in this study represents when gaze and speech focus on the same visual stimuli or story component during narration. Importantly, DCRP takes into consideration the time lag between two time series (e.g., gaze and speech on the same story component during narration)[52], which is accommodated using a sliding window. Through this approach, the DCRP analyses quantified the temporal relationship between gaze focus and speech on the same story component, and the amount of correspondence between gaze and speech in content across the narration.

DCRP analyses were conducted using the CRQA package [70] in R to characterize both the temporal relationship between gaze and speech on the same story components, and the content correspondence between gaze focus and speech content. A 10s sliding window was applied, allowing alignment of gaze and speech on the same story component within a ±10s delay.

The DCRP analysis produces a recurrence plot from which different measures of the coordination between two time series were calculated (see Figure 1). On the plot, the x-axis represents the time lag between two time series, while the y-axis represents the recurrence rate or percentage of coordination. The grey line of synchrony (LOS) represents when there is no time lag between the two time series, meaning the behaviors happen simultaneously. The primary measures used to quantify gaze-speech coordination are described below:

1. Recurrence rate (RR): Recurrence rate quantifies the proportion of recurrence/correspondence between two time series (i.e., gaze focus and narrative content examining the same story component/visual stimuli). In the recurrence plot, RR was calculated as the total area under the curve, reflecting the total amount of synchrony between gaze and speech across different time lags within the sliding window (see Figure 1). In the current study, a higher RR indicates greater consistency between gaze focus and speech content, representing greater gaze-speech content coordination.

2. Recurrence rate peak (RRpeak): Recurrence rate peak measures the highest proportion of correspondence between gaze and speech during narration, observed within the sliding time window. RRpeak is represented in the recurrence plot as the peak of the curve, and the value was calculated as the percentage of RR at the specific time lag (see Figure 1). A higher RRpeak represents a greater maximum amount of coordination across different time tags between time series, which refers to the greatest level of content coordination or consistency between gaze and speech across narration in the current study.

3. Qlos: Qlos measures the extent to which one behavioral time series leads the other, i.e., gaze leads speech on the same story components, in time. Qlos was calculated as the ratio between the area under the curve to the left of the LOS (representing the extent to which gaze was leading speech in time) and the size under the curve to the right of LOS (representing the extent to which speech was leading gaze). A higher Qlos represents a greater temporal lead of gaze over speech, or the average extent of gaze-speech temporal coordination across narration.

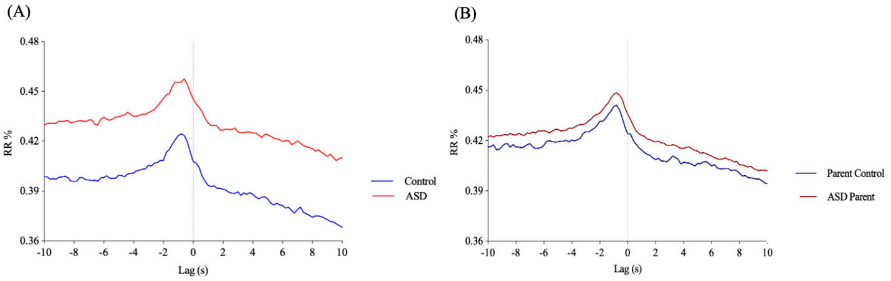

Figure S1. Gaze-speech coordination was calculated based on the DCRP plots. The temporal coordination (i.e., Qlos) was calculated by dividing size under curve on the left of line of synchrony (LOS) at lag= 0s, by that on the right of the LOS. The content coordination measures, i.e., RR and RRpeak, were calculated as the total size under the curve, and the highest peak, respectively.

Results: Correlations

Table S1. Correlations with ASD symptom severity.

Symptom Severity from ADOS	Gaze-speech Coordination	
	Qlos	RR
Overall	*r*= -.19, *p*= .32	*r*= -.03, *p*= .89
Social Affect	*r*= -.02, *p*= .92	*r*= -.20, *p*= .28
Restricted and Repetitive Behaviors	*r*= -.36, *p*= .05	*r*= .17, *p*= .37

Note. Qlos: temporal coordination, the extent to which gaze was ahead of speech in time; RR: content coordination, the total amount of content consistency between gaze and speech. * *p* < .05, ** *p* < .01, *** *p* <.001.

Table S2. Correlations with narrative quality.

Narrative Quality	ASD Group		ASD Parent Group	
	Qlos	RR	Qlos	RR
Story Components Present	*r*= .16, *p*= .39	*r*= -.09, *p*=. 65	*r*= .12, *p*= .28	*r*= -.19, *p*=. 09
Causal Inferences	*r*= -.14, *p*= .45	*r*= .12, *p*= .54	*r*= .06, *p*= .57	*r*= -.14, *p*= .20
Affect/Cognition	*r*= -.35, *p*= .06	*r*= .40, *p*=.03*, *adjusted p* = .34	*r*= .14, *p*= .19	*r*= -.11, *p*=.30
**Narrative Quality**	**ASD and Non-ASD Groups Combined**		**Parent Groups Combined**	
	Qlos	RR	Qlos	RR

Story Components Present	r= .08, p= .52	r= -.09, p=.45	r= .13, p= .16	r= -.19, p=.04*, *adjusted* p = .34
Causal Inferences	r= -.02, p= .90	r= .01, p= .93	r= .17, p= .07	r= -.18, p=.05
Affect/Cognition	r= -.01, p= .92	r= .10, p= .41	r= .10, p= .26	r= -.05, p= .59

Note. Affect/Cognition represents the percentage of descriptions of thoughts/emotions of the story characters; Story Components Present measures the inclusion of key story elements in narrative; Causal Inferences refers to the percentage of causal explanations of story events and actions of the story characters. Qlos: temporal coordination, the extent to which gaze was ahead of speech in time; RR: content coordination, the total amount of content consistency between gaze and speech. * $p < .05$, ** $p < .01$, *** $p <.001$.

Table S3. Correlations with pragmatic language ability.

	ASD Group		ASD and Non-ASD Groups Combined	
	Qlos	RR	Qlos	RR
Pragmatic Language Violations (PRS-SA Scores)	r= -.37, p= .05	r= .13, p= . 49	r= -.31, p= .03*, *adjusted* p = .08	r= .22, p=. 13
	ASD Parent Group		Parent Groups Combined	
	Qlos	RR	Qlos	RR
Pragmatic Language Violations (PRS Scores)	r= -.19, p= .09	r= .26, p= .02*, adjusted p = .08	r= -.17, p= .07	r= .26, p=.005**, *adjusted* p = .04*

Note. The Pragmatic Rating Scale-School Age (PRS-SA) and the Pragmatic Rating Scale (PRS) were used to assess conversational pragmatic language abilities. Qlos: temporal coordination, the extent to which gaze was ahead of speech in time; RR: content coordination, the total amount of content consistency between gaze and speech. * $p < .05$, ** $p < .01$, *** $p <.001$.
